# Quality of Life in Cardiovascular Surgery: Elaboration and Initial Internal Validation of a Quality of Life Questionnaire

**DOI:** 10.21470/1678-9741-2018-0108

**Published:** 2018

**Authors:** Marina Macedo Kuenzer Bond, Jenny Lourdes Rivas de Oliveira, Luiz Carlos Bento de Souza, Pedro Silvio Farsky, Vivian Lerner Amato, Dorival Julio Della Togna, Samira Kaissar Ghorayeb, Magaly Arrais dos Santos

**Affiliations:** 1 Instituto Dante Pazzanese de Cardiologia, São Paulo, Brazil.

**Keywords:** Quality of Life, Cardiovascular Surgical Procedures, Coronary Artery Bypass

## Abstract

**Objective:**

Elaboration and internal validation of the Quality of Life in Cardiovascular Surgery (QLCS) questionnaire adapted to the reality of Brazilian cardiovascular surgery.

**Methods:**

Cross-sectional pilot study of a prospective cohort included in the Documentation and Surgical Registry Center (CEDREC) for internal validation of the QLCS questionnaire. Four hundred forty-five patients submitted to cardiovascular surgery and who answered a QLCS questionnaire 30 days after hospital discharge were included. It was applied via telephone. To verify the questions' internal consistency, Cronbach's alpha was used. The total QLCS score was calculated as the sum of 5 questions, ranging from 5 to 25 points. Mann-U-Whitney test was used to relate the symptoms with the quality of life (QoL). Level of significance was 5%.

**Results:**

After 30 days of surgery, about 95% of the patients had already returned to normal routine and 19% of them were already performing physical activity. In the evaluation of the QLCS's internal consistency, a Cronbach's alpha of 0.74 was found, suggesting that this was probably an adequate questionnaire to evaluate QoL in this population. In the comparison between the presence and absence of symptoms and the median of QoL, the presence of pain at the incision (*P*=0.002), chest pain (*P*<0.001), shortness of breath (*P*<0.001), and return to physical activity (*P*<0.001) were statistically significant.

**Conclusion:**

The process of elaboration and validation of questionnaires includes a series of steps. The QLCS questionnaire is probably an adequate tool for the evaluation of QoL in the postoperative patient of cardiovascular surgery, in this first stage of internal validation.

**Table:** 

Abbreviations, acronyms & symbols	
CABG	= Coronary artery bypass grafting
CEDREC	= Documentation and Surgical Registry Center
KCCQ	= Kansas City Cardiomyopathy Questionnaire
LQ	= Lower quartile
MOS SF-36 or SF-36	= Medical Outcomes Study 36-Item Short-Form Health Survey
QLCS	= Quality of Life in Cardiovascular Surgery
QoL	= Quality of life
SF-12	= Short version of MOS SF-36 or SF-36
UQ	= Upper quartile
WHOQOL-100	= The World Health Organization Quality of Life Assessment
WHOQOL-BREF	= Summary version of WHOQOL-100
WHOQOL-group	= The World Health Organization Quality of Life Group

## INTRODUCTION

Traditionally defined by philosophers and poets, quality of life (QoL) and its measurement are becoming a fundamental topic for the practice of Medicine^[1]^.

According to The World Health Organization Quality of Life Group (WHOQOL-group), QoL is defined as an individual's perception of his/her position in life in the context of the culture and value systems in which he/she lives and in relation to his/her goals, expectations, standards, and concerns. It is a broad ranging concept affected in a complex way by the person's physical health, psychological state, personal beliefs, social relationships, and his/her relationship to salient features of his/her environment^[2]^.

The impact of any event or health status on a person's QoL depends to a large extent on his/her personal projects, desires, and history^[1]^. Although the most important indicator of health status is the clinical outcome, QoL and patient satisfaction are key indicators for the adequate understanding and treatment of pathologies^[3]^. The QoL related to cardiovascular disease and the impact of the treatment on each patient's life have been researched, contributing to the clinical decision-making process, as well as improving patient care, aiming at a more integral approach to health (physical, psychological, and social)^[4]^.

To evaluate QoL, you can choose to use generic questionnaires, which do not specify the disease itself, such as the Medical Outcomes Study 36-Item Short-Form Health Survey (MOS SF-36 or SF-36)^[5]^, and its short version 12 (SF-12)^[6]^, or The World Health Organization Quality of Life Assessment (WHOQOL-100)^[7]^, and its summary version (WHOQOL-BREF)^[8]^, or specific questionnaires, developed for a particular pathology, such as the Kansas City Cardiomyopathy Questionnaire (KCCQ) for heart failure^[9]^.

Improving QoL is one of the main objectives for cardiovascular surgery, so a review on this subject was published in 2011, which included 29 articles published between January 2004 and December 2010, but only nine studies presented data on pre and postoperative QoL, and that was a limitation^[10]^. Another meta-analysis published in 2013^[11]^ selected 15 articles on QoL in the postoperative period of cardiovascular surgery, and most of the analyzed studies showed a significant improvement in QoL in the evaluation instruments; the SF-36 was the most used questionnaire in 43% of the studies.

Therefore, QoL measurement of the postoperative period of cardiovascular surgery is crucial to evaluate the patients' treatment, and the creation and validation of a current questionnaire which is more adequate for these patients' reality is of fundamental importance. The objectives of this study were to create, based on an adaptation of the main existing QoL questionnaires, and to validate a current and adequate questionnaire, easy to apply, which can be performed via telephone, for the reality of the postoperative period of cardiovascular surgery in Brazil.

## METHODS

This is a pilot study for initial validation of the Quality of Life in Cardiovascular Surgery (QLCS) questionnaire, using as sample the population included in the Documentation and Surgical Registry Center (CEDREC) of a hospital specialized in Cardiology. CEDREC is a computerized database specifically for patients undergoing cardiovascular surgery in our hospital. Patients who were operated on and accepted to participate in the database were included and monitored prospectively.

This cross-sectional study taken from a prospective cohort included patients who underwent cardiovascular surgery, from July 2016 until October 2017, who accepted to participate in CEDREC, and who properly answered a QoL questionnaire (the QLCS) after thirty days of hospital discharge. Only patients under 18 years old were excluded. All the other patients were included in the sample.

The questionnaire was created by a group of medical specialists, based on the main existing QoL questionnaires (SF-36, SF-12, WHOQOL-100, WHOQOL-BREF), and adapted to the current reality of Brazilian medicine. It is a simple and fast questionnaire, easy to perform, and feasible to be applied via telephone. The questionnaire used by CEDREC had 21 questions (Table 1), being five questions on QoL, which correspond to the QLCS questionnaire (Table 2), and the others were about medication, symptoms, procedures, and internationalization in that period. These five QLCS questions were created and considered adequate and clear enough by all medical experts to compose a questionnaire whose main objective is to easily and quickly assess patients' QoL in the postoperative period of cardiovascular surgery. The total QoL score was calculated as the sum of the five QoL questions, ranging from 5 to 25 points. The higher the value found, the better the QoL.

**Table 1:** Complete questionnaire used by the Documentation and Surgical Registry Center (CEDREC) (English version).

This questionnaire asks for your opinion about your health and how you feel and about your ability to perform your daily activities in the last period (30 days, 6 months, 12 months, and annually).					
1) Has the patient taken any tests in the last period?					
Yes	No	Does not know	Not applicable		
2) Has the patient had any intervention in the last period?					
Yes	No	Does not know	Not applicable		
3) Has the patient had any surgical procedure in the last period?					
Yes	No	Does not know	Not applicable		
4) Is the patient taking any medication?					
Yes	No	Does not know	Not applicable		
5) Does the patient feel pain in the surgical incision?					
Ys	No	Does not know	Not applicable		
6) Has the patient had any infection?					
Yes	No	Does not know	Not applicable		
7) Does the patient feel chest pain?					
Yes	No	Does not know	Not applicable		
8) Does the patient experience shortness of breath?					
Yes	No	Does not know	Not applicable		
9) Did the patient have other symptoms?					
Yes	No	Does not know	Not applicable		
10) Does the patient do physical activity?					
Yes	No	Does not know	Not applicable		
11) Did the patient return to normal work/school/activities?					
Yes	No	Does not know	Not applicable		
12) How is the patient's performance in daily activities/work/school?					
Too bad	Bad	Good	Very good	Great	Not applicable
13) How is the patient's health after surgery?					
Too bad	Bad	Good	Very good	Great	Not applicable
14) How is the patient's physical capacity after surgery?					
Too bad	Bad	Good	Very good	Great	Not applicable
15) From the emotional point of view, how is the patient feeling?					
Too bad	Bad	Good	Very good	Great	Not applicable
16) In the relationship with his/her family members, how is the patient feeling?					
Too bad	Bad	Good	Very good	Great	Not applicable
17) Did the patient have chest trauma?					
Yes	No	Not applicable			
18) Did the patient have fainting episodes?					
Yes	No	Not applicable			
19) Did the patient feel palpitations?					
Yes	No	Not applicable			
20) Did the patient have a fall and suffer cranial trauma?					
Yes	no	Not applicable			
21) Did the patient gain or lose weight?					
Yes	NO	Not applicable			

**Table 2:** Quality of Life in Cardiovascular Surgery (QLCS) questionnaire (English version).

This questionnaire asks for your opinion about your health and how you feel and about your ability to perform your daily activities in the last 30 days, 6 months, 12 months, and annually.
1 - Too Bad 2 - Bad 3 - Good 4 - Very Good 5 - Great
1) How is the patient's performance in daily activities/work/school?
2) How is the patient's health after surgery?
3) How is the patient's physical capacity after surgery?
4) From the emotional point of view, how is the patient feeling?
5) In the relationship with his/her family members, how is the patient feeling?
Total:________/ 25 points

After the creation of any questionnaire, it must go through an initial validation step with a pilot sample. In this pilot test, the questionnaire's final version is administered to a large representative sample of respondents for whom the questionnaire is intended. At this point, it is important to evaluate the questionnaire's reliability. This is accomplished by assessing the internal consistency and testing-retesting reliability. Internal consistency reflects the extent to which the questionnaire items are intercorrelated or whether they are consistent in measurement of the same construct^[12]^. Internal consistency is commonly estimated using the alpha coefficient, also known as Cronbach's alpha^[13]^.

For statistical analysis, Cronbach's alpha was used to verify the questionnaire's internal consistency and to validate the questionnaire. Assuming a standard deviation of approximately 6 units and a detectable difference of 3 points, with 5% of alpha error and 95% of power, it would be required a total of 63 cases. The quantitative variables were described by mean and standard deviation in the presence of normal distribution, or median and interquartile range in the presence of asymmetric distribution. The qualitative variables will be presented by absolute frequencies (number of patients) and relative frequencies (percentages). The Mann-U-Whitney test was used to relate the symptoms (qualitative variable) with the QoL (quantitative variable). Level of significance was set at 5%.

## RESULTS 

Four hundred forty-five patients submitted to cardiovascular surgery, from July 2016 to October 2017, and who had a QoL questionnaire answered 30 days after hospital discharge were included. The mean age of the sample was 60.20 years (±13.29), of which 58.43% were males and 41.57% were females.

The initial characteristics of the sample are summarized in Table 3, but we can highlight that 29.6% of them were diabetic, 60% were hypertensive, 34.6% were dyslipidemic, and 28.5% had a history of smoking.

**Table 3:** Sample's characteristics.

	N	%
Female	185	41.57
Male	260	58.43
Diabetes	132	29.66
Arterial hypertension	267	60.00
Dyslipidemia	154	34.60
Smoker	127	28.53
Chronic obstructive pulmonary disease	10	2.24
Rheumatic fever	30	6.74
Chronic kidney disease	21	4.71
Arrhythmia	8	1.80
Stroke	8	1.80
	**Mean**	**SD**
Age (years)	60.2	13.29

N=absolute number; SD=standard deviation

**Fig. 1:**
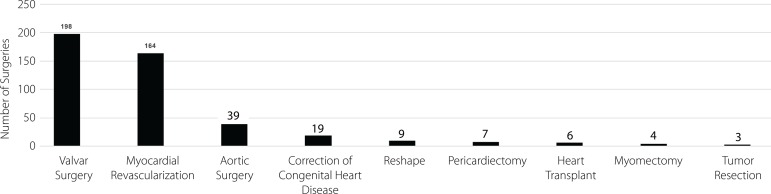
Types of surgeries performed.

About the surgeries performed, the largest part consisted of valve surgeries, with a total of 198 (44%) cases, followed by coronary artery bypass grafting (CABG) with 164 (37%) cases, aortic surgeries with 39 (8.7%) cases, and congenital anomaly correction surgery with 19 (4.2%) cases. The remainder comprised of 6 (1.3%) cases of heart transplant surgeries, 7 (1.5%) cases of pericardiectomies, 4 (0.9%) cases of myectomies, 3 (0.6%) cases of tumor removals, and 9 (2%) cases of reshaping and cleaning. It is noteworthy that in some cases more than one type was performed at the same surgery time (Figure 1).

After 30 days of surgery, 35% of the patients had incision pain, 17% wound infection, 23% chest pain, 22% lack of air, 1% fainting, 5% palpitation, and 43% other symptoms. However, 95% of the patients had already returned to normal routine and 19% were already performing physical activity.

In the evaluation of the QLCS's internal consistency, a Cronbach's alpha of 0.74 was found, proving this to be a questionnaire with adequate internal consistency. This means that, in the evaluation of the degree of variation of these questions, it was demonstrated that the items are appropriately related to each other.

**Fig. 2:**
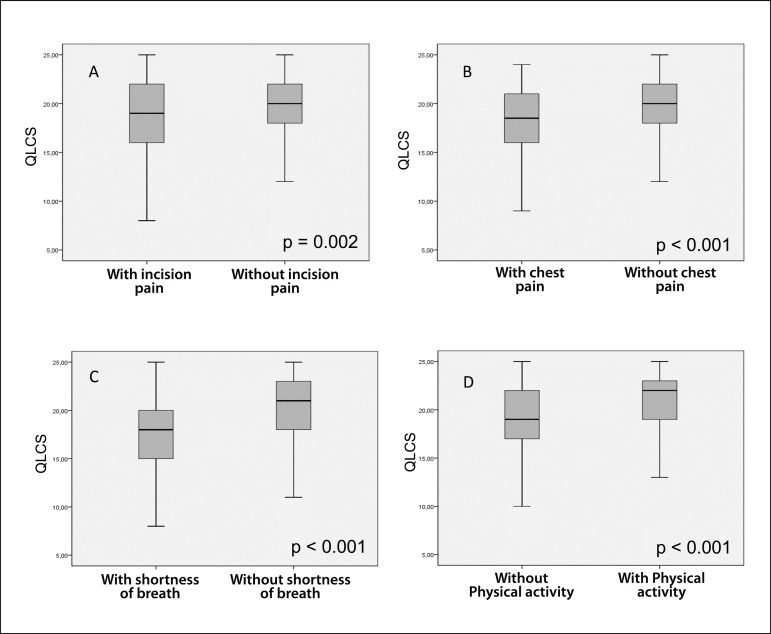
Comparison between the presence and absence of symptoms and the median obtained in the Quality of Life in Cardiovascular Surgery (QLCS) questionnaire showed in box-and-whisker plots. (A) Incision pain. (B) Chest pain. (C) Shortness of breath. (D) Physical activity.

When comparing between patients with and without incision pain after 30 days of surgery, we observed that patients with this symptom presented a median of 19.0 in the QLCS questionnaire, with lower quartile (LQ) of 16.0 and upper quartile (UQ) of 22.0, while those without incision pain presented a median of 20.0 (LQ=18.0; UQ=22). When using the Mann-U-Whitney test, this observed reduction was significant (*P*=0.002). The same was also observed for patients with chest pain, with a median of 18.50 (LQ=16.0; UQ=21.0), when comparing with those without chest pain, with a median of 20.0 (LQ=18.0; UQ=22.0); significant difference (*P*<0.001). The presence of shortness of breath also affected the QLCS score, with a median of 18.0 (LQ=15.0; UQ=20.25), compared to its absence, with a median of 21.0 (LQ=18.0; UQ=23.0); (*P*<0.001). So, in the evaluation of symptoms and QLCS scores, the presence of pain at incision (*P*=0.002), chest pain (*P*<0.001), and shortness of breath (*P*<0.001) negatively affected QoL. On the other hand, other symptoms (*P*=0.075), fainting (*P*=0.50), palpitations *P*=0.52), and infection (*P*=0.48) weren't related to QoL. The return to physical activity was also significant, the group that performed physical activity had a higher median in the QLCS questionnaire (22.0; LQ=19.0; UQ=23.0) than the sedentary group (19.0; LQ=17.0; UQ=22.0), improving QoL (*P*<0.001). When comparing the median of the group that returned to the routine (20.0; LQ=18.0; UQ=22.0) with the median of those who hadn't returned (17.0; LQ=16.0; UQ=22.0), it wasn't significant (*P*=0.85). The comparison between the presence and absence of symptoms and the median obtained in the QLCS questionnaire is shown in box-and-whisker plots in Figure 2.

## DISCUSSION

The present study represents an important advance to facilitate the assessment of QoL in postoperative cardiovascular surgery patients in Brazil and other countries. The QLCS is a new questionnaire with an internal consistency of 0.74 (Cronbach's alpha), being probably a good questionnaire to evaluate QoL in this population in a fast, easy, and objective way. Thus, it is possible to measure QoL in a practical way, even in countries where there aren't many resources and incentives for research, making possible a greater diffusion of QoL assessment by society, which contributes to the universality of knowledge. When Cronbach's is 0, it indicates no internal consistency (*i.e.*, none of the items are correlated with one another), whereas, when it is 1, it reflects perfect internal consistency (*i.e.*, all the items are perfectly correlated with one another). In practice, Cronbach's alpha of at least 0.70 has been suggested to indicate adequate internal consistency^[14]^. A low Cronbach's alpha value may be due to poor inter-relatedness between items; as such, items with low correlations with the questionnaire total score should be discarded or revised. On the other hand, an alpha value that is too high (more than 0.90) suggests that some questionnaire items may be redundant, and investigators may consider removing items that are essentially asking the same thing in multiple ways^[12]^.

The QoL in the postoperative period of cardiovascular surgery has been much studied in the international literature, which justifies even more the creation of this questionnaire. When referring to CABG, Aydin et al.^[3]^ compared pre and postoperative QoL in 120 patients over 65 years, and the SF-36 for postoperative results were higher than the SF-36 for preoperative results (*P*<0.001). The same result of improvement in QoL with CABG was also demonstrated by Dal Boni et al.^[4]^ and Gois et al.^[15]^. In another study, Takiuti et al.^[16]^ compared clinical treatment, percutaneous coronary intervention, and CABG in 483 patients with coronary disease; there was an improvement in QoL in all domains and in the three therapeutic options, but the surgery offered better results after four years of follow-up.

In a Brazilian prospective cohort study with 44 elderly patients between 60 and 80 years of age, of both sexes, diagnosed with coronary artery disease, and undergoing CABG, from June 2010 to June 2011, a significant increase was observed in SF-36 scores between the pre and postoperative periods (three and six months) for the domains: functional capacity, pain, general health, vitality, and emotional aspect.

Albert et al.^[17]^ found that in the postoperative period of surgery for valvulopathy correction, the improvement of the gradient resulted in an improvement in the QoL. Another study, conducted by Ferreira et al.^[18]^, showed that patients with cardiovascular valvar disease have a better QoL three to six months after surgery, compared to those awaiting surgical intervention.

Some studies addressed cardiovascular surgeries as a whole; a study published in 2008 by Colak et al.^[19]^, which analyzed 111 patients in pre and postoperative periods and the health status of the patients one year after discharge, showed a statistically significant improvement, and the group of high-risk patients had the best benefit (EuroSCORE 6). Koch et al.^[20]^, in a study of 5581 patients, demonstrated that a lower socioeconomic status of the patient is associated with a lower QoL, while Noyez et al.^[21]^ concluded that QoL in cardiovascular surgery is overestimated, certainly for the elderly and those patients with preoperative low QoL.

Several authors, as described above, have already performed studies about the QoL assessment in the postoperative period of cardiovascular surgery, but a Brazilian study with a questionnaire created in Brazil, adapted to our reality, hasn't been conducted yet. In addition, this questionnaire was created to facilitate the acquisition of data, not only via personal contact, but also via telephone. By facilitating the way to obtain information about QoL, more people may be interested in using this parameter in the assessment of individuals' health, especially in the Brazilian context, where research isn't valued and resources are scarce.

The present study has some limitations. The first, it is a cross-sectional analysis of a cohort. This was done only for the QLCS questionnaire's initial validation, the work with longitudinal analysis of the patients is already being elaborated and the test-retest reliability will be made. The second, it is an unicentric study. On the other hand, once validated, we hope that many other scientific centers can take advantage of this questionnaire. The third refers to the limited number of patients, however, because it is a prospective study using the CEDREC registry, which began in June 2016, it was not possible to recruit a larger sample. Still, it was enough to perform the internal validation of the questionnaire, since only 63 patients were required. A fourth limitation is the possible bias associated with any questionnaire conducted via telephone. But it was created to be simpler and more objective to reduce this bias, and all phone calls were made by the same researcher. A fifth limitation could be the fact that we haven't chosen to create stricter exclusion criteria, excluding patients with lung diseases or neurodegenerative diseases. The QoL of these individuals would be invariably affected by these other conditions, and the insertion of these patients could provide a bias for the misunderstanding of adequately representing the impact of cardiovascular surgery on QoL. However, this could be done in a future research.

Finally, this is only the first validation step of this questionnaire, which presents adequate internal consistency. Subsequent steps will be performed to ensure the adequate use in the clinical practice of postoperative cardiovascular surgery patients.

## CONCLUSION

The QLCS questionnaire is probably a good tool for the evaluation of QoL in the postoperative period of cardiovascular surgery, with adequate internal consistency, besides being an easy-to-apply instrument, which can even be done via telephone. The presence of chest pain, incision pain, and shortness of breath seems to be related to QoL, making it worse, while return to physical activity seems to improve QoL.

**Table:** 

Authors' roles & responsibilities	
MMKB	Substantial contributions to the conception or design of the work; or the acquisition, analysis, or interpretation of data for the work; drafting the work or revising it critically for important intellectual content; final approval of the version to be published
JLRO	Substantial contributions to the conception or design of the work; or the acquisition, analysis, or interpretation of data for the work; drafting the work or revising it critically for important intellectual content; final approval of the version to be published
LCBS	Substantial contributions to the conception or design of the work; or the acquisition, analysis, or interpretation of data for the work; drafting the work or revising it critically for important intellectual content; final approval of the version to be published
PSF	Final approval of the version to be published
VLA	Final approval of the version to be published
DJDT	Final approval of the version to be published
SKG	Final approval of the version to be published
MAS	Substantial contributions to the conception or design of the work; or the acquisition, analysis, or interpretation of data for the work; drafting the work or revising it critically for important intellectual content; agreement to be accountable for all aspects of the work in ensuring that questions related to the accuracy or integrity of any part of the work are appropriately investigated and resolved; final approval of the version to be published
